# Machine Learning-Based Blockchain Technology for Secure V2X Communication: Open Challenges and Solutions

**DOI:** 10.3390/s25154793

**Published:** 2025-08-04

**Authors:** Yonas Teweldemedhin Gebrezgiher, Sekione Reward Jeremiah, Xianjun Deng, Jong Hyuk Park

**Affiliations:** 1 Department of Computer Science and Engineering, Seoul National University of Science and Technology, Seoul 01811, Republic of Korea; yonas@seoultech.ac.kr; 2 Department of Electrical and Information Engineering, Seoul National University of Science and Technology, Seoul 01811, Republic of Korea; reward@seoultech.ac.kr; 3 Hubei Key Laboratory of Distributed System Security, Hubei Engineering Research Center on Big Data Security, School of Cyber Science and Engineering, Huazhong University of Science and Technology, Wuhan 430074, China; dengxj615@hust.edu.cn

**Keywords:** V2X security, machine learning, blockchain, multi-access edge computing

## Abstract

Vehicle-to-everything (V2X) communication is a fundamental technology in the development of intelligent transportation systems, encompassing vehicle-to-vehicle (V2V), infrastructure (V2I), and pedestrian (V2P) communications. This technology enables connected and autonomous vehicles (CAVs) to interact with their surroundings, significantly enhancing road safety, traffic efficiency, and driving comfort. However, as V2X communication becomes more widespread, it becomes a prime target for adversarial and persistent cyberattacks, posing significant threats to the security and privacy of CAVs. These challenges are compounded by the dynamic nature of vehicular networks and the stringent requirements for real-time data processing and decision-making. Much research is on using novel technologies such as machine learning, blockchain, and cryptography to secure V2X communications. Our survey highlights the security challenges faced by V2X communications and assesses current ML and blockchain-based solutions, revealing significant gaps and opportunities for improvement. Specifically, our survey focuses on studies integrating ML, blockchain, and multi-access edge computing (MEC) for low latency, robust, and dynamic security in V2X networks. Based on our findings, we outline a conceptual framework that synergizes ML, blockchain, and MEC to address some of the identified security challenges. This integrated framework demonstrates the potential for real-time anomaly detection, decentralized data sharing, and enhanced system scalability. The survey concludes by identifying future research directions and outlining the remaining challenges for securing V2X communications in the face of evolving threats.

## 1. Introduction

We are on the edge of a new technological era characterized by connected autonomous vehicles (CAVs), which promise to deliver unparalleled user experiences, enhanced road safety, balanced transportation environments, and applications [1]. Despite the potential benefits, the safety of driverless cars remains a paramount concern in the public view, particularly considering incidents such as the death of a pedestrian in 2018 caused by an Uber self-driving car and the Tesla car crash in 2021 [2]. The ever-changing network topology due to mobility in V2X presents a significant challenge, especially regarding security frameworks [3]. The communication link between vehicles is vulnerable to internal and/or external cyberattacks [4]; Internal cyberattacks, which are hard to detect due to their valid credentials, can compromise nodes, initiating malicious behavior. Additionally, quantum-capable devices have the potential to break encryption methods used in security conventions, exposing communications security [5]. The simulation results in Ref. [6] indicate that as the number of attacked vehicles and the severity of the cyberattacks increase, the impact on the traffic flow becomes more pronounced, with lower capacity, increased risk of rear-end collisions, more significant air pollution, and higher fuel consumption. As the deployment of CAVs increases and organizations become more aware of the threats, CAV cybersecurity will assume increasing importance. The development of new detection and assessment methods for various types of attacks, the implementation of robust defensive techniques, and technology assessment will play a significant role in enhancing cyber resiliency and improving the reliability of defensive techniques [7]. Hakeem et al. [8] suggest that authentication and privacy preservation are among the most pressing concerns in V2X communications.

Securing V2X communications is crucial for granting access to network resources, and various solutions have been proposed to address the associated security issues. These solutions can be categorized into cryptography and trust-based schemes [9]. Trust-based systems, cryptographic techniques that rely on a trusted third party to sign a certificate message and certify the identity associated with a public key, have been employed to filter out nodes compromised in sensor networks [10]. Lone et al. [11] suggested a credibility-based trust model that tracks previous node misbehaviors and filters out untrustworthy recommendations originating from malevolent nodes to enhance the security of V2X communication. Despite the widespread use of cryptography-based solutions, they may not be suitable for future V2X wireless communication. Due to the decentralized and heterogeneous nature of the V2X environment, managing and maintaining keys may be challenging [12]. Incorporating post-quantum cryptography (PQC) presents a nontrivial challenge, as it requires enormous key sizes or lengthy digital signatures that were not anticipated when V2V security standards were initially developed to be flexible [13]. Attaining a solution that satisfies the requirement of low communication overhead, real-time processing, and a high-security level while maintaining a lightweight size that can be deployed on edge devices is essential [14].

To address these challenges, integrating machine learning (ML) and blockchain has emerged as a promising approach for adaptive, decentralized, and intelligent V2X security frameworks. ML algorithms enhance security by dynamically detecting cyber threats, identifying anomalous behaviors, and optimizing real-time decision-making. AI-driven swarm intelligence enables cooperative defense mechanisms among vehicles, mitigating distributed attacks [15]. Additionally, AI-powered intrusion detection systems (IDSs) provide proactive threat monitoring by continuously learning from attack patterns [16]. Cybersecurity machine learning can dynamically detect evolving threats and ensure enhanced real-time monitoring for advanced persistent threats (APTs) [17]. On the other hand, blockchain technology ensures data integrity, access control, and privacy protection through its immutable ledger and consensus mechanisms. By eliminating centralized points of failure, blockchain prevents data tampering and unauthorized access in V2X communications. However, blockchain faces notable challenges such as scalability issues, high energy consumption, and potential privacy concerns when implemented in vehicular networks [18].

Despite the individual advantages of ML and blockchain, integrating these technologies can address their respective weaknesses while enhancing security capabilities. Blockchain can enhance ML-based security frameworks by providing decentralized trust management, improving data privacy, and enabling secure model sharing across distributed vehicular nodes. Simultaneously, ML can optimize blockchain networks by improving consensus mechanisms, reducing computational overhead, and facilitating intelligent smart contracts for efficient transaction validation [19]. While both ML and blockchain have demonstrated significant potential for V2X security, existing research lacks a holistic approach that effectively integrates these technologies to address critical security vulnerabilities such as latency constraints, real-time attack detection, and privacy preservation. Previous studies have primarily focused on either blockchain or ML individually, leaving unexplored synergies that could significantly enhance security and efficiency. Furthermore, challenges related to lightweight deployment on resource-constrained edge devices require further investigation. Thus, the main contributions of this survey paper can be summarized as listed below:

- We provide a comprehensive categorization of security threats in V2X, distinguishing between long-standing persistent issues and emerging adversarial techniques, offering a structured foundation for designing robust defense mechanisms.
- This survey presents a holistic analysis of the synergistic integration of three key technologies: ML, blockchain, and multi-access edge computing (MEC). Unlike previous works that focused on these technologies in isolation, we scrutinize how their combined strengths can address critical gaps in V2X security such as real-time threat detection, decentralized trust management, and low-latency processing.
- We present a multi-layered service scenario architecture that cohesively combines ML, blockchain, and MEC. This conceptual framework serves as a practical blueprint for future research and development.
- Based on our integrated analysis, we identify and discuss specific open research challenges and future directions that arise directly from the convergence of these technologies.

**Figure 1:**
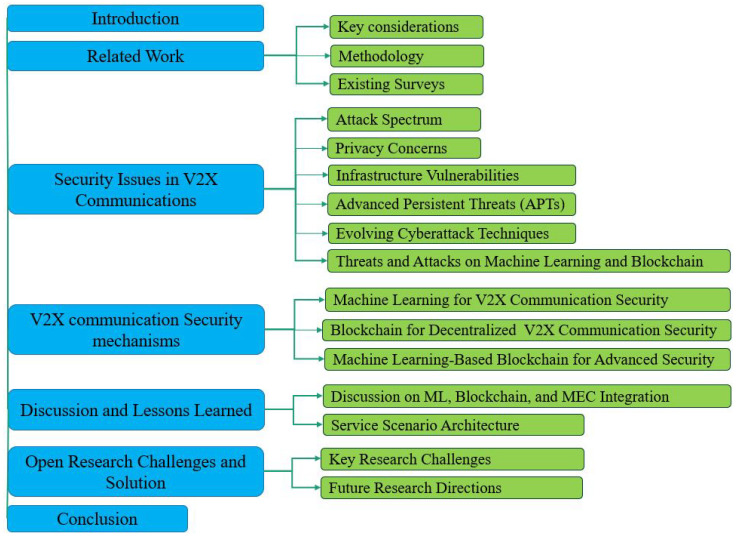
Breakdown of the survey paper structure.

As depicted in Figure 1, the remainder of the paper is structured follows. Section 2 examines related work, comparing and contrasting its key contributions and limitations to our research. Section 3 delves into persistent and adversarial security challenges in V2X communications. Section 4 discusses various security solutions using ML and blockchain technology and their limitations as presented in the literature. Section 5 elucidates the primary objective of this survey, which is the integration of ML and blockchain. Section 6 discusses unresolved challenges and future research directions. Finally, Section 7 concludes our survey paper.

## 2. Related Work

We first discuss the existing surveys on blockchain and ML in V2X communication security and contrast them with our survey. We further discuss their main contributions and limitations, and then add our contributions.

### 2.1. Key Considerations

The following are the principal considerations for our work, which differentiate and enhance our paper in comparison to the existing literature:

- Security: Security in V2X communications is defined by the CIA triad—confidentiality, integrity, and availability. Confidentiality protects sensitive information like vehicle location and user data from unauthorized access to prevent privacy breaches. Integrity ensures that the data exchanged between vehicles and infrastructure remains accurate and unaltered, maintaining trust and preventing dangerous misinformation. Availability guarantees that critical services, such as traffic control and real-time vehicle communication, are accessible when needed, preventing disruptions that could lead to accidents and traffic issues. Together, these principles uphold the security and functionality of V2X systems.
- Role of ML and blockchain: ML and blockchain serve complementary roles in meeting these security needs. Machine learning techniques bolster V2X security by enabling real-time threat detection and adaptive responses. For instance, ML can analyze vehicular data to identify anomalies indicative of cyberattacks or classify threats based on patterns, improving system resilience in dynamic environments. Blockchain provides decentralized solutions for secure data management and trust. Its tamper-proof nature ensures data integrity, while its distributed architecture supports secure identity verification and logging, reducing reliance on vulnerable centralized systems. We provide insights into utilizing blockchain technology in securing V2X communications including decentralized vehicle identity management, secure message broadcasting, and tamper-proof data logging.
- Scalability: As V2X networks grow, scalability emerges as a key challenge. We examine the role of machine (deep) learning in processing and analyzing vast amounts of vehicular data, improving threat detection, and real-time decision-making in traffic scenarios. Additionally, we discuss the challenges in integrating blockchain technology into existing V2X systems and potential solutions such as scalability and latency issues. As a solution, we discuss MEC, which enhances scalability by enabling localized data processing and reducing latency.

### 2.2. Methodology

**Figure 2:**
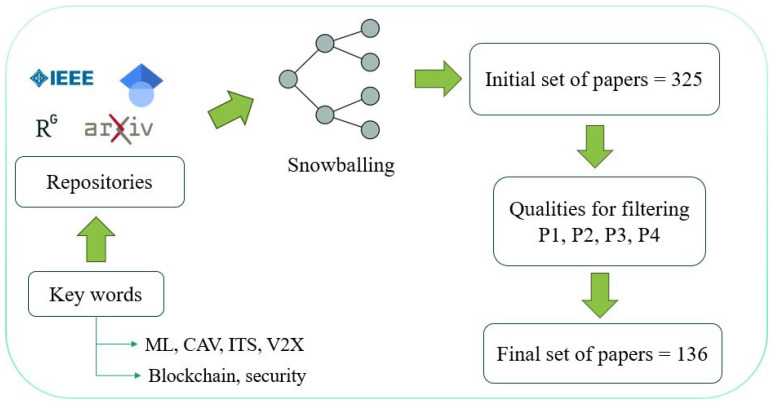
Methodology of conducting our survey.

To ensure a comprehensive and focused review of machine learning and blockchain applications in V2X communication security, we employed a systematic literature search and selection process. The process, as can be seen in Figure 2, involved a comprehensive literature search across several known repositories including Google Scholar, ResearchGate, IEEE Xplore, ACM Digital Library, and arXiv. We used a set of relevant keywords such V2X security, machine learning in vehicular networks, blockchain for Internet of vehicles (IoV), secure V2X communication protocols, anomaly detection in V2X, decentralized authentication in vehicular networks, adversarial attacks on machine learning in V2X, and blockchain scalability in IoV. After identifying an initial set of papers, we applied the snowballing method, where references and citations from selected papers were reviewed to uncover additional relevant studies. This iterative process ensured that our survey captured a broad and representative set of research on V2X communication security, highlighting key developments and trends in integrating machine learning, blockchain, and cybersecurity technologies.

- Relevance to V2X security (P1): Studies must specifically address security challenges in V2X communication systems such as data integrity, confidentiality, authentication, or availability. Papers focusing solely on general vehicular networks or non-security aspects were excluded.
- Technological focus on machine learning and blockchain (P2): Papers covering various ML techniques that addressed anomaly detection, threat identification, or network security, giving priority to studies that focused on real-time applications and adaptability in dynamic environments. Additionally, we selected papers on blockchain applications for secure data sharing, decentralized identity verification, and integrity assurance in V2X networks, especially those tackling latency and scalability in resource-constrained environments. We also placed a focus on papers exploring the integration of ML and blockchain, either in concept or in practice.

- Evaluation of capabilities and limitations (P3): We focused on studies that provided a critical assessment of each technology’s effectiveness and constraints. This included:
  ✓ Performance evaluations of machine learning models (e.g., accuracy, false positive rates);
  ✓ Analysis of blockchain’s impact on latency, computational overhead, and scalability;
  ✓ Discussion of vulnerabilities such as adversarial attacks on ML models or centralization risks in blockchain networks.
- Cutting-edge research (P4): As V2X security is a rapidly evolving area, we prioritized recent publications (last 3–5 years) to ensure that the technologies discussed were current and applicable to modern V2X systems. Moreover, we looked for studies that identified unresolved issues, practical deployment challenges, or promising directions for future research. These insights were instrumental in forming the backbone of our discussion on future research paths.

Our search and selection process yielded a total of 135 papers, which formed the basis of this survey. These studies were categorized according to their contributions to security requirements, scalability solutions, and privacy preservation in V2X systems, as outlined in Section 2.2. This categorization ensured that our review aligned with the survey’s key focus areas while providing a balanced assessment of both the strengths and vulnerabilities of machine learning and blockchain in V2X security.

### 2.3. Existing Surveys

Yoshizawa et al. [20] identified multiple shortcomings and inconsistencies in security and privacy standards related to V2X communications. By conducting a thorough root cause analysis, the authors delved into the underlying reasons for these issues, categorizing them according to their foundational causes. Alnasser et al. [21] evaluated existing security solutions within the V2X communication sphere through an exhaustive survey and the development of a taxonomy.

Authors Sedar et al. [22] presented a classification of security mechanisms, distinguishing them by their proactive or reactive defense strategies. This framework assists in evaluating the efficacy and potential drawbacks of current security measures against V2X attacks. Talpur et al. [23] offered a comprehensive survey of ML-based techniques for different security issues in vehicular networks. Their survey classified ML techniques used in vehicular network (VN) applications, explained their solution approaches and working principles for addressing security challenges, and discussed their limitations.

Rao et al. [24] explored the integration and implications of IoT, V2X, and blockchain technologies, addressing the security challenges and countermeasures within this context. They also presented significant hurdles and considerations for employing blockchain within intelligent transportation systems (BITSs). Similarly, Shrestha et al. [25] examined the advancements in V2X communications, exploring its evolution based on cellular 5G technology and non-cellular 802.11bd. They investigated the integration of blockchain in 5G-based MEC vehicular networks to enhance security, ensure privacy, and facilitate content caching.

Varma et al. [26] contributed to this discourse by scrutinizing the application of blockchain in securing software-defined vehicular networks (SDVNs), focusing on different software-defined networking (SDN) control plane architectures, security services, and the challenges they present. They summarized blockchain-based SDVNs and analyzed SDN controllers, blockchain platforms, and simulation tools for VANETs. Boualouache et al. [27] comprehensively surveyed and classified ML-based MDSs while discussing and analyzing them from security and machine learning perspectives. Highlighting open research and standardization issues, they offered recommendations for directing the development, validation, and deployment of machine learning-based MDSs.

Ahmad et al. [28] submitted a comprehensive examination of cyberattacks targeting both intra-vehicle and inter-vehicle communication systems. This review extended beyond traditional cybersecurity challenges and defenses within the CAV environment to thoroughly explore contemporary strategies employing machine learning, federated learning, and blockchain technologies to protect CAVs. The section elaborated on applying machine learning and data mining techniques in developing IDS and other countermeasures addressing these challenges. While this paper examined both ML and blockchain, the paper lacked a detailed analysis of their integrated potential. Alladi et al. [29] extended the discussion of V2X communication security by critically analyzing approximately 75 blockchain-based security frameworks designed for vehicular networks. The authors focused on their application, security efficacy, and underlying blockchain technology. Their survey delved into secure blockchain-based vehicular network applications and examined security prerequisites, potential attack vectors, and the architectural nuances of blockchain platforms including their types and consensus algorithm.

Rishiwal et al. [30] systematically analyzed privacy concerns and security threats within V2X communication including issues like data tampering, unauthorized access, DoS attacks, and Sybil attacks. This paper emphasized human-centric approaches, focusing on protecting user privacy and enhancing security within V2X communication networks in smart city environments. It introduced a blockchain-based framework designed to enhance the security and trustworthiness of V2X networks by preventing unauthorized access and addressing potential malicious actions. As mentioned by the authors, blockchain’s scalability in high-traffic V2X environments remains questionable, as delays and processing costs could impact performance.

Based on previously surveyed papers, no other survey paper has extensively covered machine learning and blockchain integration or suggested architecture for securing V2X communications. Our research aims to provide a basis for a practical case study demonstrating how blockchain can be combined with AI to detect anomalies and secure V2X communications. As seen in Table 1, our work provides a comprehensive review of security issues, ML and blockchain solutions, and a feasible architecture combining the solutions with MEC for a secure V2X communication future.

## 3. Security Issues in V2X Communications

The advent of V2X communications marks a significant step toward the realization of fully autonomous and connected vehicular networks [31]. However, this advancement presents substantial security and privacy challenges [32], particularly in 5G-enabled vehicular networks [33]. A lack of trust and acceptance, unclear responsibilities, and environmental and organizational challenges also hinder the transition to V2X [34]. This section delves into the persisting and adversarial security issues pivotal in V2X communications. Persisting security issues refer to long-standing, ongoing challenges in V2X communications stemming from the system’s inherent complexities. Persisting security issues in the vehicle-to-everything environment are ongoing challenges that must be addressed to warrant the safety and reliability of vehicular communications systems [35]. These challenges stem from the unique characteristics of V2X networks such as high mobility, dynamic network topology, and the need for real-time communication [36]. Adversarial security issues refer to the advanced threats posed by malicious actors seeking to compromise the integrity of V2X networks. As novel technologies like AI/ML and quantum computing become integrated into V2X communications, new challenges emerge, increasing the complexity of the defense mechanisms required. These issues are critical given the safety-critical nature of autonomous and connected vehicles.

### 3.1. Attack Spectrum

The V2X environment is vulnerable to various cyber threats that can jeopardize the safety and efficacy of vehicular networks. This was famously demonstrated by security researchers Charlie Miller and Chris Valasek in 2015 by remotely hacking a Jeep Cherokee [37]. As we explore the threat landscape, notable threats include:

- Spoofing attacks: Malevolent actors may feign legitimate vehicles or infrastructure components, transmitting false data to disrupt traffic flow or cause accidents [38]. GPS spoofing [39] is where attackers broadcast fake GPS signals to deceive a vehicle’s navigation system. When such an attack is successful, a vehicle’s perceived position can be offset by several kilometers, leading to misplacement on distant roads and creating severe safety risks. To enhance anti-interference and anti-spoofing capabilities, alternative or supplementary positioning systems, such as pseudolites, which are ground-based transmitters that can augment or provide a backup to traditional GNSS signals, have been explored [40]. Another type of spoofing attack by impersonating another vehicle or infrastructure unit, known as identity spoofing, can lead to unauthorized access to network resources and the dissemination of false information [41].
- Eavesdropping: Unauthorized interception of V2X communications can divulge sensitive information such as the drivers’ location and personal details [42]. A type of eavesdropping famously known as a man-in-the-middle (MitM) attack is where attackers intercept and potentially alter the communication between two parties, which can lead to misinformation or unauthorized data access [43].
- Denial of service (DoS) attacks: These attacks target overwhelming the network with excessive traffic, leading to service disruptions and impaired communication [44]. One way of doing this is jamming, intentionally sending disruptive signals to overload the communication channels, hindering the transmission of critical safety messages [45]. Another way is flooding, which overwhelms the network with high volumes of data packets, rendering the network unable to process legitimate requests [46]. It is important to highlight the significance of widespread distributed denial of service (DDoS) attacks in this context, as they emerge as critical facilitators within a 6G IoT environment, potentially giving rise to challenges related to network security, privacy, and trust [47].
- Sybil attacks: In a Sybil attack, an attacker forges multiple fake identities to gain an unfair advantage or disrupt network operations. In the V2X context [48], this could lead to falsifying vehicle positions or flooding the network with fake data, disrupting traffic management systems or causing accidents. Sybil attacks are particularly dangerous in decentralized networks and must be mitigated by robust identity verification and reputation-based trust systems.
- Replay attacks: Replay attacks involve intercepting and retransmitting legitimate communication messages to create false impressions or manipulate vehicular behavior. For example, an attacker could capture a message indicating a vehicle’s location and replay it at a different time to mislead traffic management systems or create congestion [49]. Timestamping, cryptographic hashing, and nonce-based schemes can help mitigate these attacks by ensuring message freshness and authenticity.

### 3.2. Privacy Concerns

Another persisting concern is privacy. Privacy in V2X communications is a vital issue that requires striking a delicate balance between efficient traffic management and the protection of personal data [50]. This becomes particularly complex due to the vast amount of data generated and shared among vehicles, roadside units, and infrastructure components. Several key challenges must be addressed:

- User anonymity: Ensuring user anonymity in vehicle-related data is a complex challenge, as highlighted by several studies. Hara et al. [51] and Troncoso et al. [52] both emphasized the need for effective methods to reduce traceability and protect privacy in location-based services and V2X communication. Pseudonymization and frequent ID changing are two potential strategies, but they need to be carefully implemented to prevent attackers from linking pseudonyms back to real-world identities.
- Data aggregation risks: Aggregating vehicular data over time can inadvertently disclose patterns that might lead to user identification. This re-identification risk is influenced by the background knowledge of potential attackers, with a trade-off between privacy and data utility [53]. This necessitates the deployment of privacy-preserving algorithms like differential privacy or homomorphic encryption to balance data utility with privacy.

### 3.3. Infrastructure Vulnerabilities

Infrastructure vulnerability is a notable persistent security issue for V2X systems. The current V2X infrastructure, which often employs centralized models, presents several vulnerabilities [54] such as:

- Single points of failure: Although V2X aims to decentralize communications, many systems still rely on centralized control mechanisms. Centralized systems can become prime targets for cyberattacks, potentially impacting traffic engineering, network design, and system reliability [55]. Centralized architectures, such as those used in traffic management systems or cloud-based services, can create vulnerabilities where a single point of compromise can impact the entire network.
- Distributed elements as targets: The decentralized nature of V2X systems, while essential for supporting low-latency communications, also introduces multiple points of vulnerability. Roadside units (RSUs) and MEC nodes may be susceptible to physical tampering or remote cyberattacks, compromising the integrity and security of the entire network. Attackers could target these elements to introduce malware, intercept communications, or alter traffic management data [56].
- Scalability issues: As the number of connected vehicles grows, the existing infrastructure may face challenges in efficiently managing and allocating resources, such as bandwidth and computing power, leading to traffic overload and energy consumption [57].

To mitigate these risks, blockchain-based distributed trust models and MEC security frameworks can be employed to decentralize trust and reduce the risk of single points of failure. Additionally, edge-based IDS and secure communication protocols between RSUs and MEC nodes can help detect and prevent intrusions in real-time.

### 3.4. Advanced Persistent Threats (APTs)

The characteristics of APTs have been described by Al-Matarneh et al. [58]. These prolonged, covert, and aimed at compromising network security represent sophisticated and prolonged attacks in V2X communications. APTs are a significant concern in the V2X communications environment as they are sophisticated, well-resourced, and specifically target high-profile entities [59]. These attacks are slow-moving and aim to quietly compromise interconnected information systems, often to steal sensitive data [60]. They are challenging to detect and handle because they employ diverse attack techniques [61]. Traditional security mechanisms are often insufficient to prevent these targeted attacks, highlighting the need for advanced security services [62].

As presented in Table 2, APTs have the ability to undermine all three CIA principles, making them among the most, if not the most dangerous threat, to V2X communication security. Therefore, advanced security services, such as AI-based anomaly detection and blockchain-powered authentication mechanisms, are needed to identify and counteract these threats. In addition, behavior-based detection models can continuously monitor network traffic for anomalies that might signal an APT. By correlating real-time data from vehicles, RSUs, and MEC nodes, these systems can more accurately identify subtle signs of an ongoing APT such as unusual data patterns, lateral movement, or unauthorized access attempts.

### 3.5. Evolving Cyberattack Techniques

Adversaries persistently refine their tactics to overcome existing security measures. AI-driven attacks and zero-day exploits are becoming more prevalent in V2X networks. AI and machine learning techniques, typically used to defend systems, are now being exploited by attackers to create more sophisticated and adaptive attacks.

- AI-driven assaults: Attackers increasingly use AI to craft more intelligent and dynamic threats. For instance, AI-based malware can learn from defense mechanisms and alter its behavior to evade detection. At the same time, adversarial machine learning can manipulate AI models used in V2X networks, leading to incorrect classifications or false alerts. This underscores the need for adversarial resilience in AI models, which can identify and defend against malicious inputs designed to compromise their integrity. The employment of AI by malevolent actors can result in increasingly sophisticated and responsive cyber threats [63]. The threats mentioned above can be further exacerbated by using AI, as discussed by Aliman et al. [64] in the context of AI and virtual reality.
- Zero-day vulnerabilities: Attackers may capitalize on undiscovered weaknesses in V2X technologies before they are detected and rectified [65]. Traditional defense mechanisms are often ineffective against these attacks, making it crucial to develop new detection and prevention mechanisms [66]. These vulnerabilities are particularly dangerous in V2X environments, where using novel communication protocols and emerging technologies introduces new attack surfaces. Moreover, if the security challenges posed by the malicious application of AI are not adequately mitigated, the increasing inclination toward integrating AI/ML in securing V2X communications might decline [22].
- The emergence of quantum computing presents a substantial risk to the cryptographic standards currently employed in V2X communications [67,68,69,70]. Quantum computers have the potential to break widely used encryption schemes such as RSA and elliptic curve cryptography, which are foundational to securing V2X systems. Quantum computing’s ability to process data at an exponential rate and its potential to crack digital encryption [67,70] necessitate the development and adoption of post-quantum cryptography [68]. While the threat is urgent, it is also manageable. The development of quantum-resistant cryptographic schemes is crucial to address this risk [69].

### 3.6. Threats and Attacks on Machine Learning and Blockchain

Machine learning and blockchain play critical roles in securing V2X communications, with ML enabling real-time threat detection and blockchain providing decentralized trust. However, their integration introduces distinct vulnerabilities. ML faces threats like adversarial attacks, where manipulated inputs such as falsified sensor data deceive models, jeopardizing systems like collision avoidance; data poisoning, which taints training data to reduce accuracy; and model inversion, exposing sensitive data to attackers. Blockchain, meanwhile, is susceptible to 51% attacks, where adversaries seize network control to alter transactions; smart contract flaws, exploitable to disrupt authentication; and Sybil attacks, where fake identities weaken consensus. Both technologies are vulnerable to shared threats such as DoS attacks, overwhelming system resources, and AI-driven assaults, where ML exploits blockchain weaknesses. These attacks threaten the confidentiality, integrity, and availability of V2X networks, potentially leading to misinformed decisions or system-wide failures.

## 4. V2X Communication Security Mechanisms

The safety and efficiency of CAVs depend on the security of V2X communications, as highlighted by several studies [71,72,73,74]. This section investigates the application of machine learning and blockchain technologies to enhance the security of V2X communications, examining their impacts and potential synergistic integration.

### 4.1. Machine Learning for V2X Communication Security

Machine learning, primarily through real-time anomaly detection, offers dynamic and adaptive methods to enhance security in V2X environments. Beyond real-time monitoring and pattern recognition, these systems can perform predictive analytics [75] by predicting potential vulnerabilities and threats, analyzing historical data, and enabling proactive security measures. Swessi et al. [76] and Boualouache et al. [27] emphasized the importance of ML-based IDSs and MISBEHAVIOR DETECTION SYSTEMS in minimizing V2X communication attacks. These systems rely on the availability of relevant network traffic logs and the development of effective ML-based MDSs.

Early approaches utilized both supervised and unsupervised models to identify potential threats. For instance, foundational research often employed classical ML algorithms like support vector machines (SVMs) [77] and random forests (RFs) to classify network traffic, frequently evaluating these models on general-purpose cybersecurity datasets, such as CICIDS2017, or within simulation environments like SUMO and Veins, due to the initial scarcity of V2X-specific security datasets [78].

To counter more sophisticated and coordinated cyberattacks, the research community has increasingly adopted deep learning and reinforcement learning (RL) techniques. These advanced models can capture complex, nonlinear patterns indicative of malicious behavior. Prathiba et al. [79] employed a hybrid deep anomaly detection (HDAD) approach that combined multi-agent reinforcement learning (MARL) and maximum entropy inverse reinforcement learning (MaxEntIRL) to detect anomalies in autonomous vehicles, reportedly achieving a higher accuracy than existing systems. The authors in Ref. [80] introduced the Efficient Trajectory Anomaly Detection and Classification (ETADC) framework for autonomous vehicles in 6G-V2X environments. The novelty lies in using the deep deterministic policy gradient (DDPG) algorithm to analyze multiple strategies like driving speed, distance, direction, and time.

Further pushing the architectural boundaries, Min et al. [81] developed the denoising variational transformer (DVT), an unsupervised model that incorporates a novel variational attention mechanism to better learn the distribution of normal sensor data. A key contribution of the DVT framework is its focus on interpretability, as it includes a residual interpreter designed to explain why a sample is flagged as anomalous by identifying the specific sensor channels responsible. These deep learning models, while powerful, are known to be vulnerable to adversarial examples—subtly modified inputs designed to deceive them [82].

Reinforcement learning, in particular, offers a path toward creating self-improving security systems that adapt without predefined threat signatures. Huang et al. [83] proposed a time-series anomaly detector powered by a recurrent neural network (RNN) that uses RL to learn from its experiences. Building on this concept, Sedar et al. [84] demonstrated the effectiveness of an RL-based approach for detecting misbehavior, such as false data injection attacks, by analyzing the real-time position and speed patterns of vehicles. While powerful, RL faces several challenges such as data efficiency [85], partial observability [86], high-dimensional spaces, and reward design. These issues can slow down learning, increase computational costs, and lead to suboptimal or unintended behaviors.

A further evolution in defense mechanisms involves using generative models to anticipate novel threats. Shafique et al. [87] employed ML techniques to enhance security against both AI-generated and traditional cyberattacks. The study utilized a conditional tabular generative adversarial network (CTGAN) to generate AI-based network traffic for training detection models, alongside traditional traffic patterns. By training a random forest (RF) model on this hybrid dataset, the system demonstrated high accuracy in detecting anomalies within the controller area network (CAN) standard of in-vehicle networks, achieving an accuracy of 0.93 for traditional attacks and 0.89 for AI-based attacks.

An alternative, more direct approach uses the GAN itself as the anomaly detector. Devika et al. [88] introduced VADGAN, an unsupervised GAN framework where the generator, built on an LSTM backbone, is trained to reconstruct normal vehicle behavior. Anomalies are then detected when the model fails to accurately reconstruct unseen malicious data, a failure measured by reconstruction error. Moreover, generative AI models can simulate attack patterns to train the network’s defense mechanisms to detect and mitigate potential threats effectively [89]. However, these models come with notable weaknesses. These include high computational costs, producing flawed outputs if the training data are biased or incomplete, training instability, and lack of interpretability [90].

In different approaches to enhance detection, Aziz et al. [91] developed an explainable neural network (xNN) model for detecting cyberattacks, specifically DoS, on the IoV. The model demonstrates high accuracy in classifying attacks on vehicular networks, outperforming other deep learning models like CNN, LSTM, and DNN in datasets like UNSW-NB15 and CICIDS2019. Oleiwi et al. [92] proposed an ensemble learning algorithm-based anomaly detection in communication networks (EL-ADCNs) that involved preprocessing, feature selection using a hybrid method (CFS–RF), and hybrid ensemble learning algorithms for intrusion detection. The system was tested on three datasets, achieving high accuracy and low false alarm rates.

In addition to detection, to address the critical need for data privacy, recent work has focused on decentralized learning paradigms [93]. Boualouache et al. [94] proposed a novel multi-process federated learning (FL) approach to detect network slicing attacks in 6G-V2X environments. Their method trains separate machine learning models for each network slice in different mobile network operators (MNOs), ensuring isolation and privacy. Once trained, these models are combined using stacking, a technique that creates a unified global model, enabling effective cross-border attack detection. To protect privacy, differential privacy is applied to the global model, adding noise to prevent data leakage. However, the system faces limitations, such as reduced performance when dealing with non-independent and identically distributed (non-IID) data across network slices, an accuracy trade-off due to differential privacy and increased training time.

Yakan et al. [95] introduced an FL-based system for detecting misbehavior in 5G vehicle-to-network (V2N) services. The approach demonstrated high effectiveness, achieving 98.4% accuracy, 99.3% precision, and a 96.9% detection rate, suggesting its practical applicability in enhancing 5G V2N service security. FL faces significant security risks, particularly from malicious clients who can launch poisoning attacks by submitting manipulated gradients or incorrect model parameters, ultimately degrading the performance of the global model [96]. Additionally, the central server, responsible for collecting and aggregating these parameters, creates a single point of failure. If this server is compromised or malfunctions, the entire system could collapse, leading to widespread disruptions.

While these advanced architectures demonstrate high efficacy, their computational complexity presents a significant barrier to deployment on resource-constrained V2X hardware like OBUs and RSUs. To address this challenge, research has focused on model lightweighting. Huang et al. [97] proposed UltraADV, a framework designed to produce lightweight but powerful anomaly detectors using knowledge distillation. In this approach, a large, complex teacher model, a transformer-LSTM autoencoder is first trained to achieve high performance. Its learned knowledge is then transferred to a much smaller and more efficient student model, which can be deployed in the real world. The UltraADV framework demonstrated that a lightweight student model could achieve an F1-score of 0.9811 while reducing the number of parameters by over 82% and the prediction time by over 60% compared with its teacher. The datasets, specific machine learning algorithms used by the methods mentioned in this subsection, including their intended purpose and their reported effectiveness, are summarized in Table 3 below.

### 4.2. Blockchain for Decentralized V2X Communication Security

Blockchain technology offers a decentralized and tamper-resistant ledger, making it an ideal candidate for enhancing security in V2X communications [98]. Zrikem et al. [99] introduced a novel architecture that leverages blockchain technology for secure and decentralized interactions in intelligent transportation systems. This architecture emphasizes the technical implementation of smart contracts and proposes a decentralized payment system and marketplace, enhancing data integration, validity, and secure messaging in smart cities.

#### 4.2.1. Decentralization of Trust and Immutable Ledger

Blockchain’s trust management enables a trustless environment where transactions and communications can be verified without a central authority [100]. Blockchain technology can potentially revolutionize the V2X environment by providing a trustless, secure, and efficient platform for data sharing and communication. For example, Smartverse [101], a blockchain-based authentication method, has been proposed to validate messages in V2X communications, addressing the security and privacy risks associated with V2X applications. Rasheed et al. [102] further enhanced this by using blockchain for trusted authentication and service awareness in 5G and beyond V2X communication, ensuring security and privacy. Arora et al. [103] introduced a blockchain-inspired lightweight trust-based system in vehicular networks, eliminating the need for third-party verification and enhancing security and privacy. Similarly, the paper by Ma et al. [104] reinforces this by proposing a distributed authentication system for IoV through smart contracts and the practical byzantine fault tolerance (PBFT) consensus algorithm, ensuring that each vehicle’s authentication result is securely added to the blockchain ledger.

A core security benefit of blockchain is its immutable ledger, which ensures data integrity and non-repudiation. Once a transaction or message is validated and recorded on the chain, it cannot be retroactively altered or deleted, providing a verifiable and trustworthy audit trail for all V2X communications [18]. Despite the benefits of blockchain’s immutability, there are also concerns about its impact on privacy laws and illegal content, leading to the proposal of a concept for a mutable blockchain structure that maintains verifiability [105]. This is crucial for accountability in safety-critical events and is highlighted by Rawat et al. [106] as a mechanism for transparent record-keeping. Additionally, a remote verification security model based on a privacy-preserving blockchain for V2X was proposed, enhancing the security of intelligent vehicle communications [107].

This immutability is the foundation for frameworks like the one proposed by Jabbar et al. [108], PSEV where parking payment transactions are broadcast and locked into a block, rendering them permanent and indisputable. However, the very permanence of the ledger creates its own challenges, particularly in relation to privacy laws like GDPR, which include the “right to be forgotten.” This has sparked debate and led to proposals for concepts like verifiable mutability, which aim to allow for data modification under strict, auditable conditions while maintaining the chain’s overall integrity.

Blockchain’s decentralized nature using a distributed ledger ensures no single point of failure, enhancing the robustness of the communication system. This is achieved by utilizing a robust, resilient, and reliable architecture that harnesses mobile edge computing and blockchain [109], a blockchain-based IoT security solution that establishes trust and continuous security [110], and the integration of blockchain with 5G MEC to address security vulnerabilities [111].

#### 4.2.2. Scalability and Security Challenges in V2X Blockchains

Blockchain’s inherent latency remains a challenge in real-time V2X environments, particularly when using consensus mechanisms like proof of work (PoW), which are computationally intensive and slow. Recent advancements such as proof of stake (PoS) and sharding can offer more scalable and efficient solutions [112]. PoS reduces the computational overhead by selecting validators based on their stake in the system rather than relying on complex calculations. Sharding improves blockchain’s scalability by splitting the ledger into smaller fragments, allowing for parallel transaction processing, which can significantly reduce latency in V2X systems.

Moreover, off-chain solutions [113] like the lightning network or state channels can help mitigate the limitations of traditional blockchain mechanisms by moving certain transactions off the main blockchain. These solutions are particularly beneficial for V2X communications, where low-latency and high throughput are critical. Off-chain mechanisms ensure that minor data exchanges (e.g., between vehicles and roadside units) are handled efficiently, reducing the bottleneck caused by on-chain validation.

Furthermore, while the ledger itself is immutable, the broader blockchain system is susceptible to unique systemic vulnerabilities. A 51% attack [114], for instance, occurs when a malicious entity gains control of the majority of the network’s hashing power or validation stake, enabling them to manipulate new transactions or halt operations. While the decentralized nature of V2X can mitigate this risk by distributing trust, it remains a concern. To counter this, modified consensus mechanisms like the two-step verification proof of stake (PoS) system proposed by Khatoon et al. [115] are being developed to prevent any single entity from gaining majority control.

Another significant vulnerability lies within smart contracts. Flaws or bugs in their code can be exploited by attackers to trigger unintended actions, tamper with data, or disrupt services, creating a critical failure point even if the underlying blockchain remains secure. Rigorous code audits and formal verification techniques are therefore essential before deploying smart contracts in safety-critical V2X applications.

### 4.3. Machine Learning-Based Blockchain for Advanced Security

Integrating synergistic ML and blockchain technologies can offer a comprehensive and robust security framework for V2X communications.

#### 4.3.1. Integrated Architectures for Proactive Defense

The integration of ML and blockchain offers a synergistic security framework where each technology compensates for the other’s limitations. For example, Zamanirafe et al. [116] highlighted how IoV applications benefit from the fusion of these two technologies, particularly in addressing key challenges such as data privacy, scalability, and security. They proposed a multi-tier hierarchical architecture where ML algorithms are applied in real-time to process data collected from IoV devices such as vehicles and roadside units (RSUs). At the same time, blockchain provides a decentralized, tamper-proof infrastructure that ensures the security and integrity of this data.

This synergy is especially prominent in federated learning applications. For example, the blockchain-based federated extreme learning machine (BEF-IDS) system introduced in [117] uses blockchain to create a secure, immutable record of shared training models, ensuring the integrity of the collaborative intrusion detection process. This prevents malicious participants from poisoning the global model with corrupt updates.

Another powerful synergy uses ML as an intelligent gatekeeper to filter and classify data before it is processed or stored on the blockchain. This enhances network efficiency and security by preventing malicious or unnecessary data from cluttering the ledger. Jadav et al. [118] showcased this approach with an architecture that used AI classifiers, such as random forest, to distinguish between attack and non-attack data in V2X communication. Benign data are then securely processed through a garlic routing network and encrypted, while the transactions are managed by a blockchain-integrated gateway. This ensures that the blockchain is primarily used to secure legitimate and verified information.

Building on this, other proposed frameworks have integrated these technologies with 6G and edge computing to further enhance performance. Nair et al. [119] developed a system using a Gaussian naive Bayes classifier to identify security threats with high accuracy, leveraging the InterPlanetary File System (IPFS) and a 6G network to overcome the limitations of traditional in-vehicle CAN protocols. A similar architecture in [120] also used a random forest algorithm for data classification alongside edge nodes and IPFS to reduce latency and improve scalability, demonstrating the practical benefits of combining AI’s real-time analytics with blockchain’s distributed trust on next-generation networks.

#### 4.3.2. AI-Driven Optimization of Blockchain Networks

ML algorithms can help dynamically update and maintain blockchain networks, ensuring they remain resilient against evolving cyber threats. By integrating ML into blockchain-based V2X systems, it is possible to optimize resource allocation, predict traffic congestion, and detect potential threats before they impact the network. For instance, ML techniques can dynamically adjust block sizes or optimize node participation based on vehicular speed and network congestion, ensuring seamless V2X communication [121]. This is especially relevant for handling the high volume of real-time data generated by autonomous vehicles, RSUs, and edge nodes. Furthermore, Tanwar et al. [122] and Solanki et al. [123] highlight the potential of ML techniques such as SVM, clustering, bagging, and deep learning algorithms in analyzing and mitigating attacks on blockchain networks. These techniques can also be applied to optimize the consensus process, making blockchain network management more adaptive to the real-time demands of V2X systems. For example, in a congested urban environment, ML algorithms can prioritize critical messages, such as collision alerts, while delaying less urgent data transactions. This ability to dynamically manage traffic loads in blockchain networks is critical to ensuring the real-time responsiveness required by autonomous vehicles [124].

Additionally, by utilizing FL, V2X systems can maintain data privacy while still benefiting from collective learning across multiple vehicles. This integration ensures that the data used for training ML models remain secure and private, aligning with the privacy requirements of V2X networks. He et al. [125] proposed Bift, a decentralized machine learning system that integrates federated learning (FL) with blockchain to provide privacy-preserving and resilient ML for CAVs. In Bift, vehicles train ML models locally using their own driving data, which are then shared through a blockchain-based consensus mechanism called proof of federated learning (PoFL). This approach enhances the scalability and robustness of blockchain-based V2X systems by ensuring that CAVs can participate in secure and decentralized model training without relying on a central server.

The PoFL consensus algorithm aggregates local models while resisting malicious attacks, providing a robust defense against data poisoning and ensuring the integrity of the global model. By leveraging Byzantine-robust aggregation rules, Bift can mitigate the impact of malicious clients that may attempt to upload incorrect model parameters. Additionally, IPFS-based off-chain storage is used to efficiently handle large datasets, further enhancing the performance of federated learning in distributed environments. However, despite the advantages of Bift, some challenges remain. The computational overhead introduced by Byzantine-robust aggregation rules and the latency caused by the PoFL consensus mechanism can hinder real-time performance, especially in high-density environments where quick decision-making is essential. Furthermore, while the system is resistant to malicious attacks, managing the complexity of local model aggregation across distributed nodes can introduce additional coordination overhead, particularly as the number of participants grows.

In a similar vein, Hai et al. [126] proposed a games-based CIA security mechanism for IoV systems, integrating blockchain and AI technologies. Their framework utilizes physical unclonable functions (PUFs) for authenticating vehicles, a flexible proof of work (dPoW) consensus mechanism, and AI-enhanced duel gaming for enhanced security. This multi-layered approach ensures the vehicular communications’ confidentiality, integrity, and authenticity, addressing issues such as side-channel and physical cloning attacks. The proposed framework was shown to outperform existing systems in terms of computational efficiency and security, making it a valuable contribution to the management and optimization of blockchain networks in V2X environments.

## 5. Discussion and Lessons Learned

This section outlines the advanced integration of ML and blockchain with MEC for V2X communication security, addressing current challenges and proposing directions for future research to continually enhance the security and efficiency of V2X communication systems. First, we discuss the rationale behind incorporating MEC into our conceptual architecture. Subsequently, we elaborate on the specifics and operational flow of our suggested service scenario architecture.

### 5.1. Discussion on ML, Blockchain, and MEC Integration

The key findings of this survey indicate that ensuring robust security in V2X communications requires an integrated approach that combines ML, blockchain technology, MEC. ML offers advanced capabilities for real-time anomaly detection and misbehavior detection, blockchain provides a decentralized and tamper-proof framework for secure data sharing, and MEC enhances processing efficiency by bringing computation closer to the network edge. The synergy between these technologies is crucial for addressing the complex and evolving security challenges in V2X environments. MEC offers the advantage of processing data closer to the network edge, reducing latency, and improving response times in real-time traffic management and autonomous vehicle decision-making [24].

Vladyko et al. [127] highlighted the potential of this integration by presenting a mathematical model and energy-efficient algorithm for traffic offloading. They proposed a blockchain–MEC V2X system that reduced the percentage of blocked tasks, improved average latency efficiency, and enhanced energy efficiency by significant average percentages compared to with traditional V2X systems. This implies that a blockchain-based MEC system allows RL agents to offload computation tasks to edge servers securely. Manale et al. [128] further supported this by proposing a blockchain-based approach to secure 5G-V2X communication. Cao et al. [129] discussed the integration of MEC and blockchain, emphasizing the role of public blockchain networks in providing trust and security enhancement. Moreover, in cooperative learning, blockchain can provide a trusted platform for agents to exchange knowledge, while MEC enables fast, distributed computation.

Decentralized architectures such as blockchain also present certain characteristics that—if not properly accounted for—might ultimately impinge upon the users’ privacy [130]. Accordingly, decentralized infrastructures intended to promote individual privacy, and autonomy might be much more vulnerable to governmental or corporate surveillance than their centralized counterparts. Notable research by Zhang et al. [121] introduced a cooperative learning with deep compression (CLDC) framework that integrated ML, blockchain, and MEC to optimize resource allocation and secure communications in the IoV environment. Their approach uses dueling deep Q-learning (DDQL) to dynamically adjust resource distribution based on changing network conditions such as vehicular speed and traffic congestion. This dynamic resource management enhances the real-time performance of V2X systems by reducing the time and computational overhead typically associated with traditional resource allocation methods. Moreover, regarding security, the framework also employs a learning-based redundant Byzantine fault tolerance (L-RBFT) consensus mechanism, which ensures fault tolerance and secure communications across IoV nodes. Integrated with ML, this consensus mechanism helps detect and mitigate potential cyber threats in real-time, offering a more resilient approach to blockchain network management. Additionally, by leveraging deep compressed learning in a distributed setting, the framework ensures that data privacy is preserved, aligning with the privacy-preserving requirements of V2X communication systems.

### 5.2. Service Scenario Architecture

**Figure 3:**
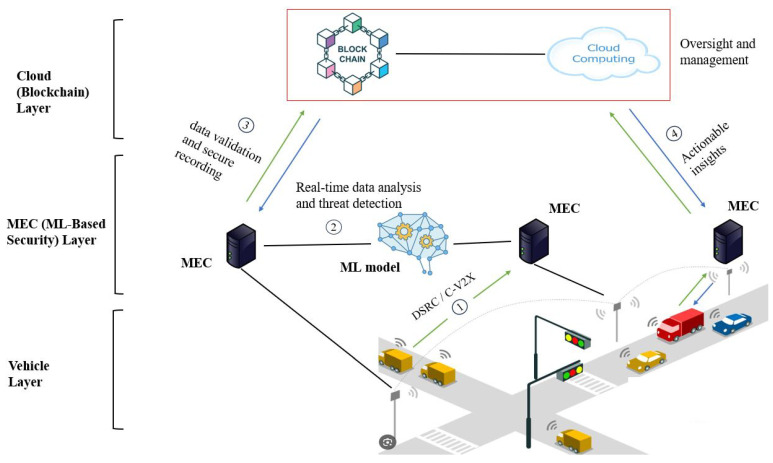
Service scenario architecture: ML, blockchain, and MEC integration.

Based on our findings, we present a conceptual architecture, as depicted in Figure 3, that integrates ML, blockchain, and MEC, serving as a future research direction for V2X security. Our conceptual framework’s combined use of these technologies ensures rapid data processing and decision-making, heightened security, and data integrity. This integration enables real-time anomaly detection, predictive analytics, and decentralized, immutable data management, which is crucial for V2X security [25].

- Vehicle layer: This is the foundational layer, consisting of vehicles equipped with various sensors and communication modules (e.g., dedicated short-range communications (DSRC) or cellular V2X (C-V2X)) that facilitate V2X communication. The modules are responsible for collecting critical data such as vehicle location, speed, and proximity to obstacles, and for communicating this information with other vehicles and nearby infrastructure. Vehicles in this layer can also be configured to host lightweight blockchain nodes, allowing them to participate directly in the decentralized ledger and enhance data authenticity at the source.
- Edge layer (MEC): The edge layer comprises MEC nodes, strategically positioned near road infrastructure (e.g., traffic lights, road signs), which serve as the computational backbone of the architecture. These nodes are equipped with advanced software modules including a data processing module for real-time data ingestion, an ML inference engine running models for anomaly detection and traffic pattern analysis, a model update manager employing federated learning for continuous adaptation, and a communication interface using encrypted protocols (e.g., TLS). MEC nodes reduce latency and facilitate immediate responses to security threats, enhancing vehicular network safety and efficiency. Supporting this layer are RSUs, which act as communication hubs using DSRC or C-V2X to relay data from vehicles to MEC nodes and participate as lightweight blockchain nodes for local validation. The integration is achieved via a blockchain gateway module, which encrypts and forwards processed data to the cloud layer, ensuring seamless interoperability between the MEC and RSU functionalities.
  ✓ ML-based security services: The architecture’s security management component leverages ML algorithms for swift threat identification and response coordination. These algorithms are distributed across the network, where MEC nodes run lightweight ML models (e.g., convolutional neural networks (CNNs) for image-based anomaly detection) to process data locally, enabling immediate responses like alerting drivers or adjusting signals.
  ✓ Blockchain network executes heavier ML analytics (e.g., LSTM networks for predictive trends) on aggregated data, ensuring tamper-proof threat logging and policy enforcement via smart contracts. The integration is facilitated by a security orchestration module on MECs, which coordinates ML outputs with blockchain transactions, ensuring prompt action (e.g., triggering cybersecurity protocols) by interfacing with vehicle control units and infrastructure systems.

- Central cloud/control center: This layer handles complex processing, long-term data storage, and system management. It employs high-performance computing resources to update ML models with global threat intelligence using batch learning, maintain blockchain protocols by adding new nodes and refining consensus algorithms, and oversee the entire operational domain by providing essential oversight and management functions. The blockchain layer operates as a decentralized ledger integrated with the MEC layer to ensure data integrity, traceability, and non-repudiation across the V2X network. This integration is achieved at a high level through the following mechanism:
  ✓ An interlayer communication where MEC nodes act as blockchain clients, submitting validated data to a distributed network of nodes (vehicles, roadside units (RSUs), and traffic management centers) via a consensus protocol that is optimized for low-latency V2X requirements.
  ✓ The blockchain serving as a data storage of critical V2X data such as transaction logs with timestamps and digital signatures, anomaly reports, and security policies (smart contract definitions enforcing access control and response actions).
  ✓ The self-executing smart contracts, written in languages like Solidity, automate security policies based on ML insights. For example, a smart contract might trigger a traffic signal adjustment if an MEC node detects a collision risk, ensuring rapid, trustless enforcement.
  ✓ The decentralized ledger is maintained across a heterogeneous network of nodes. Vehicles contribute lightweight nodes for data validation, RSUs serve as intermediate nodes for regional consensus, and traffic management centers act as full nodes for global oversight, balancing scalability and security.
- Data flow and processing mechanism: The operational flow, as indicated by the numbered steps in Figure 3, begins with raw sensor data being collected by vehicles. (①) These data are transmitted via DSRC or C-V2X to nearby edge nodes for real-time processing using embedded ML models. In the next step (②), the ML models analyze the data for anomalies or threats. These processed data and any detected anomalies or threats are then securely sent to the blockchain layer at step (③) via the blockchain gateway module. Smart contracts on the blockchain can be used to implement security policies automatically, and ML models at the central data center assess data over time, refining predictions and identifying emerging threats. The models are continuously enhanced with new data and threat intelligence. At the same time, the blockchain network is meticulously maintained and updated to include new nodes, improve consensus mechanisms, and enhance security protocols. Finally, at step (④), processed and analyzed data are communicated back to the vehicles and infrastructure. Here, vehicles and traffic systems receive actionable insights for immediate response or adjustments. The central control center oversees the entire v2x system architecture. This comprehensive architecture encapsulates a dynamic, adaptive, and secure approach to V2X communication, ensuring the reliability and robustness of these increasingly critical systems.
- Empirical validation and performance benchmarking: The architecture outlined, at present, is conceptual. A critical direction for future research is its empirical validation through realistic simulations. Joint simulation platforms, such as integrating the network simulator NS-3 with the autonomous driving simulator CARLA, would be essential for evaluating the real-world performance of the integrated ML, blockchain, and MEC framework.

## 6. Open Research Challenges and Solution

### 6.1. Key Research Challenges

The integration of ML, blockchain, and MEC offers innovative solutions to enhance the security of V2X communication systems. This integrated approach promises to address critical challenges in V2X communication by leveraging the strengths of each technology. ML provides intelligent data analysis and threat detection, blockchain ensures secure and transparent data management, while MEC facilitates efficient, low-latency data processing near the source. However, despite these advantages, several research challenges are unresolved.

- Scalability and efficiency: Key among these challenges is the issue of scalability and efficiency, particularly in densely populated vehicular environments where system demands are significantly higher [34]. As V2X networks expand and the number of connected vehicles increases, the system must efficiently manage larger volumes of data while maintaining low latency. The integration of ML and blockchain introduces computational overhead, which can become a bottleneck in dense vehicular environments. For instance, blockchain consensus mechanisms, such as PoW or PoS, require substantial computational resources, while ML models used for real-time threat detection demand significant processing power [116]. Privacy-preserving ML models, such as those using federated learning, still face issues in terms of computational load and efficient training across heterogeneous nodes in V2X environments.
- Ensuring interoperability: Ensuring interoperability poses another significant hurdle, necessitating the development of universal standards and protocols that enable seamless communication between diverse technologies, manufacturers, and service providers [131]. The V2X environment involves diverse manufacturers, devices, and communication protocols. Ensuring interoperability between these systems is essential for seamless communication between vehicles, RSUs, and infrastructure elements. The lack of universal communication protocols leads to fragmentation across vehicle manufacturers, limited RSU and infrastructure integration, and difficulties in blockchain adoption. When different companies implement proprietary V2X communication standards, it creates compatibility issues. Many existing ITS do not support cross-vendor interoperability, reducing the effectiveness of large-scale deployments. Moreover, without standardized APIs and cross-chain interoperability, integrating blockchain into existing V2X architecture remains challenging.
- Data privacy: Another critical concern is data privacy [20]; balancing the need for extensive data sharing and processing with the privacy rights of individual drivers and compliance with regulatory standards is paramount. In V2X systems, sensitive data such as driver identity, location, and movement patterns must be protected, but at the same time, these data are crucial for system-wide functionality and safety. While strong encryption and privacy-preserving ML techniques (e.g., homomorphic encryption, secure multi-party computation) can protect data, these approaches introduce increased computational complexity, which can hinder real-time decision-making. They can also create challenges in regulatory compliance, as different regions such as GDPR in Europe and CCPA in the U.S. impose strict rules on personal data usage.
- Sophisticated cyber threats: The rising sophistication of cyber threats, including zero-day exploits and APTs, which traditional security models struggle to detect and respond to in real-time. Other persisting threats include attackers who can manipulate ML models by injecting misleading data, leading to incorrect threat assessments. Blockchain 51% attacks and smart contract exploits, where flawed contract codes are exploited for financial or data breaches, are persisting sophisticated cyber threats.
- Post-quantum threats: The seemingly inevitable arrival of quantum computing presents a future challenge to the blockchain’s current cryptographic standards, calling for proactive research and development in quantum-resistant cryptographic methods [132]. While lattice-based cryptography and multivariate polynomial cryptography offer potential solutions, they introduce larger key sizes and higher computational overhead. Moreover, updating cryptographic standards across a decentralized V2X network will require gradual, large-scale deployment efforts.

### 6.2. Future Research Directions

As we look toward the future of V2X communication security, the integration of ML, blockchain, and MEC holds immense potential to address the critical challenges outlined in Section 6.1. However, realizing this potential requires targeted research to overcome scalability limitations, interoperability barriers, privacy concerns, sophisticated cyber threats, and the looming risks posed by quantum computing. This section proposes key research directions to tackle these challenges, ensuring that V2X systems are secure, efficient, and scalable for widespread adoption.

- Optimizing scalability and efficiency: The computational overhead of ML and blockchain in dense vehicular environments poses a significant bottleneck to scalability and efficiency. Future research should focus on developing lightweight ML models and efficient blockchain consensus mechanisms tailored for V2X networks. For ML, techniques such as model compression, pruning, or adaptations of federated learning could reduce the processing demands while maintaining real-time performance for threat detection and data analysis. For blockchain, exploring resource-efficient consensus protocols can minimize latency and energy consumption, enabling high throughput in large-scale V2X deployments.
- Ensuring interoperability across diverse systems: The diversity of manufacturers, devices, and protocols in the V2X communication environment demands seamless interoperability, which remains hindered by the lack of universal standards. Research efforts should prioritize the development of standardized protocols and open-source frameworks to enable cohesive communication across vehicles, RSUs, and infrastructure elements. Creating standardized APIs and communication interfaces will bridge the gap between proprietary systems, fostering a unified V2X environment that supports scalability and collaboration among stakeholders. Moreover, cross-industry collaboration is needed to establish universal V2X communication protocols that support ML-blockchain integration.
- Enhancing data privacy while enabling functionality: Balancing the need for data sharing with privacy protection is critical for user trust and regulatory compliance in V2X systems. Future research should explore advanced privacy-preserving techniques, such as differential privacy, homomorphic encryption, or secure multi-party computation, specifically designed for vehicular networks. These methods can safeguard sensitive data, such as driver identity and location, while preserving the functionality required for safety features like collision avoidance and traffic optimization. Tailoring these techniques to the decentralized nature of V2X environments will be a key focus.
- Fortifying against sophisticated cyber threats: Research should concentrate on developing AI-powered threat intelligence systems capable of real-time anomaly detection and rapid response to emerging threats. Additionally, automated patching mechanisms should be designed to swiftly address vulnerabilities across the network, leveraging MEC for decentralized, edge-based threat mitigation. These solutions must be scalable and efficient to operate in dynamic, high-stakes vehicular contexts.
- Preparing for post-quantum security challenges: Proactive research is needed to develop and implement PQC algorithms to ensure long-term security against quantum threats. The focus should not only be on developing new algorithms, but also on their practical integration into the V2X architecture. For instance, lattice-based cryptography has emerged as a promising candidate, offering signature schemes like the Bimodal Lattice Signature Scheme (BLISS) [133], which have been designed for efficiency on resource-constrained devices. However, integrating these schemes is not trivial. They often involve trade-offs, such as larger key sizes or a higher computational overhead compared with current elliptic curve cryptography. Future research must therefore analyze these performance impacts within the V2X context, ensuring that PQC solutions do not compromise the low-latency requirements of safety-critical applications. A gradual, large-scale deployment strategy will be essential for a smooth transition before quantum threats become prevalent.
- Explainable AI (XAI) for V2X security: Future systems must not only detect threats, but also provide transparent and human-understandable reasons for their decisions. Research into explainable AI (XAI) is needed to ensure that security alerts are trustworthy and auditable. This is crucial in V2X environments, where false positives can cause unnecessary traffic disruptions and false negatives can be catastrophic.
- Governance and liability in decentralized systems: The decentralized nature of the proposed architecture raises complex questions of governance and liability. Future research must address the challenges of establishing clear accountability frameworks. For instance, who is responsible if an autonomous security decision triggered by an ML model and executed via a smart contract leads to an accident? Developing robust governance models for these automated systems is critical.

## 7. Conclusions

The landscape of intelligent transportation is on the verge of a transformation, marked by the rapid evolution of CAVs, which requires resilient V2X communications security. The intricate web of interactions between vehicles, infrastructure, and pedestrians introduces unprecedented efficiencies and safety benefits, but also creates a fertile ground for cyber threats that endanger both property and human lives. This survey has meticulously navigated the convergence of ML and blockchain technologies, underpinned by MEC, to offer a conceptual method for safeguarding these critical systems.

Our analysis revealed that a standalone security solution is insufficient for the dynamic and demanding V2X environment. Instead, the integration of ML’s intelligent threat detection, blockchain’s decentralized trust and data integrity, and MEC’s low-latency edge processing provides a robust, dynamic, and resilient framework. This synergy is crucial for addressing the spectrum of security challenges, from real-time anomaly detection to ensuring the immutability of shared data, thereby fostering a reliable and secure network for all users.

The outlined ML-based blockchain architecture embedded within the MEC framework is presented not merely as a theoretical construct, but as a feasible blueprint to reshape the future of vehicular communication. It emphasizes the critical need for real-time processing, scalability, and privacy-preserving mechanisms—all essential components in the rapidly evolving landscape of autonomous transportation. However, the road ahead demands concerted research efforts to resolve persistent challenges. Successfully addressing the issues of scalability in dense networks, ensuring interoperability across diverse platforms, and fortifying systems against emerging threats, including those from the emergence of quantum computing, will be necessary. It is through tackling these future frontiers that the full potential of secure, intelligent, and autonomous transportation will be realized.

## Figures and Tables

**Table 1:** Comparison of the existing related survey papers and our survey paper.

Paper	Year	V2X Security Issues	AI/ML Based Solutions	Blockchain-Based Solutions	ML and Blockchain Integrated	Conceptual Architecture
[20]	2023	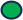	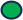	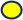	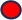	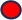
[21]	2023	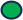	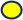	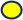	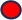	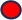
[22]	2023	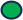	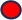	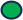	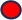	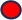
[23]	2023	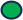	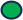	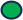	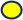	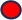
[24]	2022	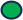	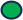	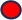	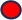	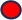
[25]	2020	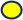	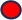	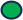	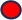	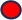
[26]	2023	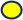	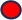	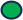	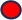	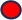
[27]	2019	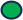	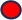	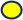	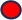	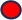
[28]	2024	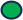	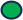	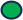	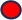	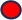
[29]	2022	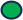	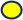	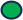	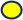	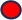
[30]	2024	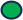	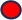	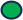	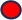	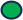
Our work	2025	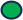	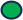	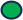	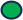	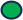

Key: 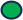: The work provides extensive coverage of the topic. 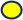: The subject is partially covered. 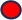: The reviewed work did not cover the subject/topic.

**Table 2:** Summary of the key security challenges and the CIA principles compromised.

Subcategory	CIA Undermined	Severity Level and Potential Damage
Spoofing attacks	Confidentiality, Integrity	False vehicle information, accidents, traffic disruption, and unauthorized access to infrastructure.
Eavesdropping (MitM)	Confidentiality	Disclosure of sensitive information, manipulation of messages, leading to security breaches.
Denial of service (DoS)	Availability	Disruption of critical communication, potentially causing accidents or halting traffic flow.
Sybil attacks	Integrity	Disrupts network integrity, leading to traffic mismanagement or collisions.
Replay attacks	Integrity	Misdirects traffic flow or vehicle behavior, potentially causing accidents or traffic jams.
Privacy concerns	Confidentiality	Tracking of vehicle movements, exposure of personal information, and user identification.
Infrastructure vulnerabilities	Availability, Integrity	Disruption of traffic systems, altered communication signals, or shutdown of critical services.
Advanced persistent threats (APTs)	Confidentiality, Integrity, Availability	Data breaches or loss of control in V2X networks through long-term, sophisticated attacks.
AI-driven assaults	Integrity	Incorrect vehicle decisions, false alerts, or system malfunctions causing accidents or system breakdowns.
Zero-day vulnerabilities	Integrity, Availability	Exploitation of unknown vulnerabilities for major system failures or data breaches.
Quantum computing threats	Confidentiality, Integrity	Compromise of entire V2X security infrastructure, allowing unauthorized access to secure data.

**Table 3:** ML algorithms for anomaly and misbehavior detection in V2X communication.

Ref.	Machine Learning Algorithm	Service in V2X Security	Dataset	Effectiveness (Metrics)
[77]	SVM	Sybil attack detection in vehicle networks	SUMO simulated urban scenario	High true positive rate of 97% with low false positive and negative rate, less than 8%.
[79]	HDAD (MARL + MaxEntIRL)	Anomaly detection	Simulated 6G V2X using NS-3 and SUMO	98% accuracy, 95% recall, and a 4% false prediction rate
[80]	DDPG	Trajectory anomaly detection	OMNET++ and SUMO (trajectory dataset)	Achieved 97% accuracy in anomaly detection and classification
[81]	DVT and variational attention mechanism	Interpretable, unsupervised anomaly detection	Simulated (Carla) and real-world vehicle dataset	F1-score of 0.9096 on real AV data and 0.8350 on simulated data
[83]	RNN (LSTM) + RL (Q-learning)	Anomaly detection	Yahoo benchmark datasets	Near 100% accuracy and recall in detecting anomalies.
[84]	RL	MDS	VeReMi dataset	Precision up to 100% and recall up to 99.79%
[87]	RF + CTGAN	IDS for in-vehicle networks	Car-hacking dataset from HCRL lab	Achieved 0.93 accuracy for traditional traffic and 0.89 accuracy for AI-generated attack detection
[88]	GAN with LSTM backbone	Unsupervised anomaly detection	VeReMi extension dataset	Achieved 81.8% overall recall with 100% recall on 5 of the 11 attack types
[91]	xNN + K-means clustering	Anomaly detection	UNSW-NB15 and CICIDS2019 dataset	Above 99% accuracy on both datasets
[92]	RF + SVM	Anomaly detection	NSL-KDD dataset	99.6% accuracy and 0.004 false-alarm rate
[93]	Decentralized federated learning (DFL)	Network anomaly detection while preserving privacy	AWID-3 dataset	Achieved a median accuracy of 71.01% across all clients, an average improvement of 19.17% over localized training
[94]	FL with stacking + DP	IDS in 6G-V2X network slices	5G-NIDD dataset	Up to 92% precision, 92% recall, and 91% f1-score
[95]	FL + LSTM	MDS	VeReMi-extension dataset	Achieved 98.4% accuracy, 99.3% precision, and 96.9% detection rate
[97]	Autoencoder and (transformer-LSTM)	Lightweight, unsupervised anomaly detection	VeReMi-extension dataset	0.9811 F1-score reducing parameters by 82% and prediction time by 62%

## Data Availability

There is no data reported.

## Abbreviations

The following abbreviations are used in this manuscript:

APTs	Advanced persistent threats
CAVs	Connected and autonomous vehicles
CNN	Convolutional neural network
CAN	Controller area network
CIA	Confidentiality, Integrity, and Availability
C-V2X	Cellular vehicle-to-everything
DSRC	Dedicated short-range communications
FL	Federated learning
IDS	Intrusion detection system
IoV	Internet of vehicles
IPFS	InterPlanetary File System
ITS	Intelligent transportation systems
MEC	Multi-access edge computing
ML	Machine learning
PBFT	Practical Byzantine Fault Tolerance
PQC	Post-quantum cryptography
PoS	Proof of stake
PoW	Proof of work
RF	Random forest
RL	Reinforcement learning
RNN	Recurrent neural network
RSU	Roadside unit
SDN	Software-defined networking
SDVNs	Software-defined vehicular networks
SVM	Support vector machine
V2X	Vehicle-to-everything
